# The Balance between Hydrogen Bonds, Halogen Bonds, and Chalcogen Bonds in the Crystal Structures of a Series of 1,3,4-Chalcogenadiazoles

**DOI:** 10.3390/molecules26144125

**Published:** 2021-07-07

**Authors:** Viraj De Silva, Boris B. Averkiev, Abhijeet S. Sinha, Christer B. Aakeröy

**Affiliations:** Department of Chemistry, Kansas State University, Manhattan, KS 66506, USA; slvdesilva@ksu.edu (V.D.S.); averkiev@ksu.edu (B.B.A.); sinha@ksu.edu (A.S.S.)

**Keywords:** chalcogen bond, halogen bond, hydrogen bond, intermolecular interactions, polymorphism, σ-hole

## Abstract

In order to explore how specific atom-to-atom replacements change the electrostatic potentials on 1,3,4-chalcogenadiazole derivatives, and to deliberately alter the balance between intermolecular interactions, four target molecules were synthesized and characterized. DFT calculations indicated that the atom-to-atom substitution of Br with I, and S with Se enhanced the σ-hole potentials, thus increasing the structure directing ability of halogen bonds and chalcogen bonds as compared to intermolecular hydrogen bonding. The delicate balance between these intermolecular forces was further underlined by the formation of two polymorphs of 5-(4-iodophenyl)-1,3,4-thiadiazol-2-amine; Form I displayed all three interactions while Form II only showed hydrogen and chalcogen bonding. The results emphasize that the deliberate alterations of the electrostatic potential on polarizable atoms can cause specific and deliberate changes to the main synthons and subsequent assemblies in the structures of this family of compounds.

## 1. Introduction

One of the main goals of crystal engineering is to employ intermolecular interactions in the assembly of crystalline materials with desired physiochemical properties [1,2,3]. To realize this objective, and to effectively manipulate these synthetic vectors, we need to further improve our understanding of the nature of these interactions.

The most influential and well-understood intermolecular force is, undoubtedly, the hydrogen bond [4], and, consequently, the active use of this supramolecular tool has been extensively mapped out [5,6,7]. Hydrogen bonding as a structure directing force has found many practical applications in pharmaceutical co-crystallizations [8,9], new agricultural formulations [10], as well as in the design of new energetic materials [11]. However, over the past few decades, the recognition and understanding of potentially competing intermolecular interactions, such as halogen and chalcogen bonding, have opened alternative ways for bottom-up design of new crystalline materials [12,13,14].

**Scheme 1:**
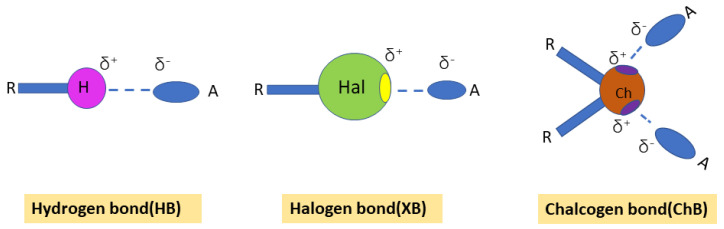
A simple view of the electrostatic component of hydrogen, halogen, and chalcogen bonds.

Hydrogen bonding is a noncovalent interaction involving the positive electrostatic potential on the hydrogen atom and a negative electrostatic potential of the acceptor atom (Scheme 1). Electrostatic models can predict the directionality of the hydrogen bond reasonably well; however, consideration of other components, such as charge transfer and dispersion, is needed to properly understand the directionality [15,16]. Like hydrogen bonds, halogen bonds share some of the same characteristics, whereby a positive electrostatic potential on a halogen atom and the negative electrostatic potential on an acceptor atom can produce a ‘halogen bond’. In fact, this interaction is even more directional than the hydrogen bond; however, it is worth keeping in mind that halogen bonds are not purely electrostatic. Using high-level theoretical models, such as symmetry-adapted perturbation theory (SAPT), it is possible to obtain the breakdown of the intermolecular forces into individual components of electrostatic, polarization, repulsion, and dispersion for a complete understanding of the forces at play, which shows electrostatics alone cannot completely describe and predict the structure directing ability of non-covalent interactions [17,18]. SAPT calculations and other periodic DFT calculations can be computationally taxing, hence one of the simplest ways to estimate the directionality of such systems with acceptable accuracy is through the use of molecular electrostatic potential maps (MEPs). This approach provides a simple method for mapping out the distribution of electron densities in molecular systems (in vacuo), whereby electron-efficient and -deficient areas can be identified [19,20]. The most prominent σ-holes (electron-deficient areas) of a molecule can be identified by the positive electrostatic potential. The σ-hole on halogen atoms provides the main electrostatic driver for halogen bonding interactions [21,22].

Chalcogen bonds, a relatively recent ‘discovery’, also share the same aspects of the halogen bond, including the high directionality. However, chalcogen atoms can form two highly directional chalcogen bonding interactions as a result of the presence of two σ-holes directly opposed to R-Ch bonds (Scheme 1) [23,24,25,26]. Importantly, by changing the electrostatic potentials on these donor atoms, these noncovalent interactions can be fine-tuned to be strong structure directing tools that can be utilized in crystal engineering for creating competition between hydrogen, halogen, and chalcogen bonding [27,28].

**Figure 1:**
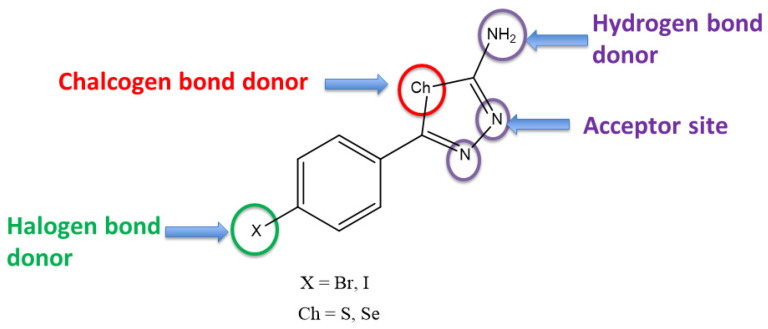
1,3,4-Chalcogenadiazole derivative with multiple donor and acceptor sites.

Many drug molecules contain multiple functional groups comprising hydrogen, halogen, and chalcogen atoms, and the presence of these can lead to increasing complexity as to how molecules interact not only in drug formulations, such as co-crystals, but also at an active site of specific receptors [8,29]. Each approved drug costs billions of dollars in research and development and many potentially useful candidates fail due to low solubility or poor stability, and such problems may be addressed with a more complete understanding of how the balance between intermolecular forces leads to a specific structure with unique bulk properties. Furthermore, 1,3,4-chalcogenadiazole moieties are known to show anti-inflammatory and anticancer properties [30,31], and broadly speaking, chalcogen atoms and their structural influence can also positively impact synthetic transformations and catalysis. Given the fact that chalcogenadiazole moieties contain a variety of domains that can act as chalcogen-bond donor/acceptor sites, they present a unique source for the formation of a multitude of intermolecular interactions. The balance and competition between these non-covalent interactions will significantly impact solid-state structure and subsequently the properties of the resulting bulk solid. In addition, the presence of chalcogen bonds can, in principle, compete with or disrupt an intended supramolecular synthetic strategy. These increased complexities can give rise to unexpected or unpredictable structural influences, hence a systematic study of the possible structure directing interactions of these moieties are required. Herein, we present a systematic structural study on how the increased complexity of multiple competing intermolecular forces in a single molecule is manifested in the solid-state. We focus on the 1,3,4-chalcogenadiazole moiety (Figure 1) with different substituents in the second and fifth position as this allows us to alter the chalcogen-bond donor from S to Se and the halogen-bond donor from Br to I, allowing us to explore the balance between these interactions. Such atom-to-atom substitutions can potentially result in significant changes to the properties, such as, for example, increased binding affinity of halogenated ligands I > Br [32], as well as increased catalytic activity of selenocysteine-containing proteins over cysteine [33,34], which could be associated with the shorter halogen or chalcogen bond formation in biological systems. In TTF-TCNQ semiconductor systems, replacement of S with Se results in *M-I* transitions at lower temperatures due to stronger coupling between the donor and acceptor [35]. With an improved insight into how chalcogen atoms form intermolecular interactions, we may be able to (i) deepen our understanding of the origins of photophysical behavior of charge-transfer complexes and (ii) develop more effective bottom-up syntheses for new classes of semi-conductors and other high-value molecular materials. 

**Figure 2:**
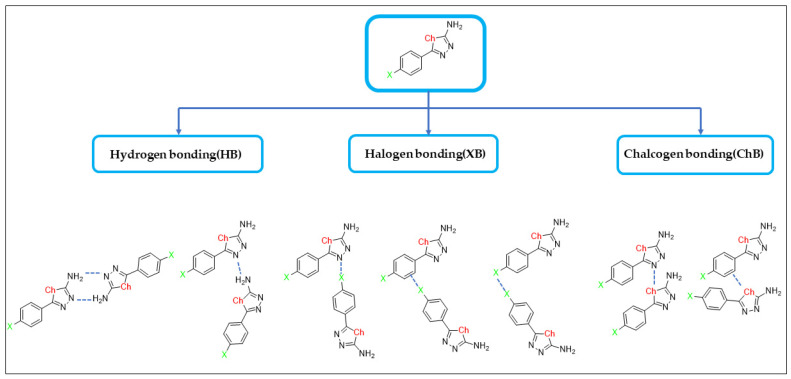
Postulated interactions in the solid-state landscape of 1,3,4-chalcogenadiazoles.

Based on relevant structures in the CSD [36,37,38,39], as well as on preliminary hydrogen-bond propensity calculations (Table S1, Scheme S1) [40], it was deemed likely that a dimer formation through a pair of identical hydrogen bonding (hydrogen-bond dimer) would be most prominent in this system. However, the tunability of the different interactions that can be produced within single component systems (Figure 2) can, in principle, lead to a range of different synthons and structural arrangements. In this study, we wanted to map out the structural landscape of a series of 1,3,4-chalcogenadiazoles and address the following specific questions: (i) To what extent do atom-to-atom replacements change electrostatic potentials? (ii) Which structure directing synthons are formed and how do they control assembly? and (iii) Can the balance between hydrogen bonds, halogen bonds, and chalcogen bonds be deliberately modulated?

## 2. Results

**Figure 3:**
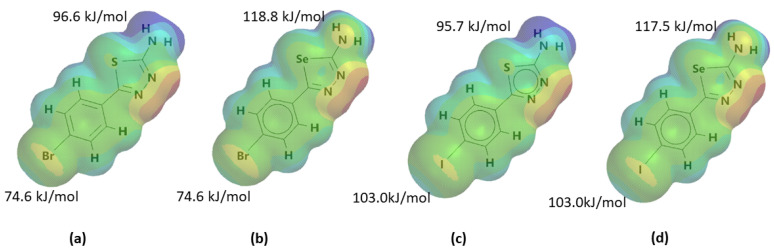
Molecular electrostatic potential maps (MEPs) of the (**a**) T1-S-Br (**b**) T1-Se-Br (**c**) T1-S-I (**d**) T1-Se-I.

The four target molecules are shown in Figure 3. Molecular electrostatic potential (MEP) maps (in vacuo) of these molecules reveal how the magnitude of the MEPs change in response to atom-to-atom replacements. A summary of the results is given in Table 1. Based on these calculations, the positive electrostatic potentials on the more polarizable atoms were observed to be larger, as expected. Electrostatic potentials on the same atom type on different analogs did not change considerably when atom-to-atom replacements were done.

**Figure 4:**
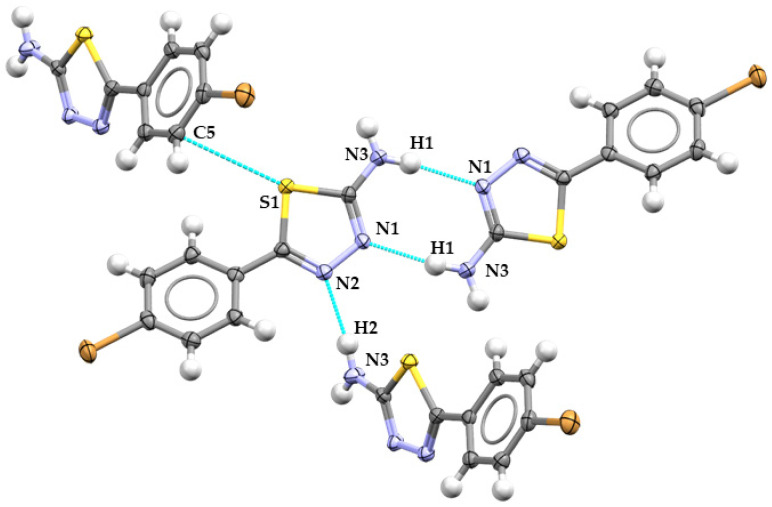
Structure directing interactions in the crystal structure of T1-S-Br [41].

The crystal structure previously reported of T1-S-Br, CCDC refcode XUVTAK [41], showed the hydrogen-bonded dimer formation with N3-H1···N1 and a single hydrogen bond between N3-H2···N2 (Figure 2, far left schematic). The bromine atom did not participate in any notable halogen bonds; however, the sulfur atom engaged in a chalcogen bond with the π-electron cloud, S1···C5, at 3.497 Å and 161.20° (Figure 4).

**Figure 5:**
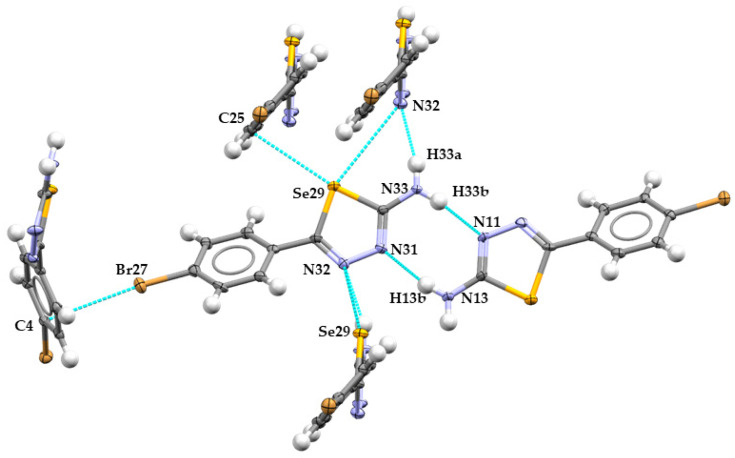
Primary intermolecular interactions in the crystal structure of T1-Se-Br.

The crystal structure of **T1-Se-Br** contains two unique molecules in the asymmetric unit with each of them showing different interactions with respect to the surrounding molecules. The halogen atom in one of them formed a near-linear halogen bond (Figure 5), Br···π electron cloud, Br27···C4 at 3.517(6) Å and 170.0(2)°, while the other formed an irregular Br7···Br27 contact in an almost perpendicular fashion, 99° (Figure S1). The selenium atoms formed a bifurcated chalcogen bond, Se···π electron cloud and Se···N (Figure 5, Figure S1).

**Figure 6:**
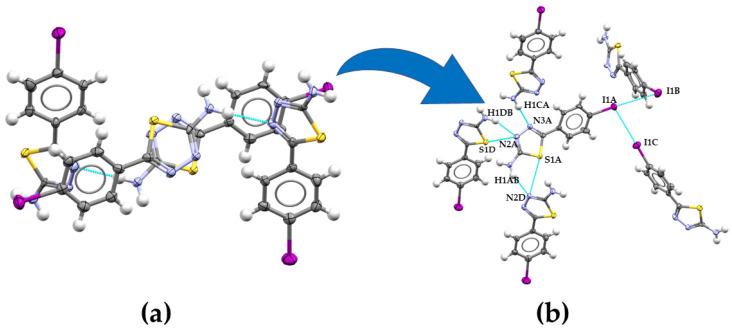
**(a**) Asymmetric unit and (**b**) main intermolecular interactions in one of the four unique molecules in the crystal structure of T1-S-I, Form I.

**T1-S-I** crystallized in two different polymorphs. Form I was obtained from acetonitrile, and Form II from methanol. The polymorphs had different numbers of symmetry-independent molecules (Z’) and completely different intermolecular connectivity. Form I resulted in Z’ = 4, where all of them displayed closely related intermolecular connectivity but with different bond geometries (Figure S2). Two molecules in the asymmetric unit formed Type II halogen bonds that formed with the electrophilic area on one halogen atom with an electron-rich area on the other halogen. In this halogen bonding, the halogen atom simultaneously acted as both the donor and acceptor and the other two unique molecules did not show any halogen bonds. The sulfur atoms engaged in chalcogen bonds between S···N in which the nitrogen atom acted as a chalcogen-bond acceptor as well as a hydrogen-bond acceptor, indicating that the hydrogen bond was most likely the prominent interaction. In addition, two hydrogen bonds per molecule were formed between NH···N (Figure 6), leading to a 2-D chain-like assembly (Figure S3).

**Figure 7:**
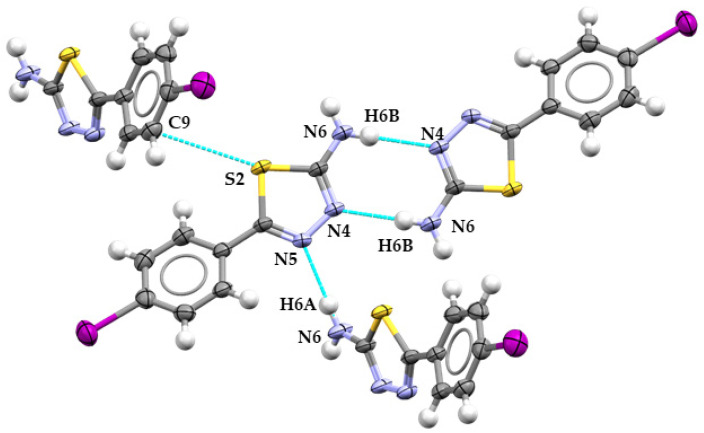
Primary intermolecular contacts in the crystal structure of T1-S-I, Form II.

The crystal structure of Form II (Figure 7) contains a hydrogen bond dimer between N6-H6B···N4 and a single hydrogen bond between N6H6A···N5 of the heterocycle moiety. No halogen bonds were present in the Form II (Figure 7). Finally, a chalcogen bond comprised of S···π ring, S2···C9, at 3.568(7) Å and 162.1(2)° was observed.

**Figure 8:**
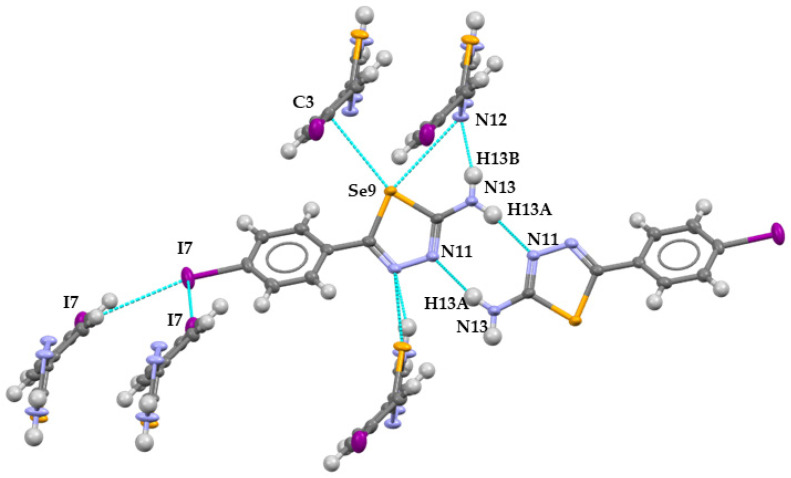
Structure directing interactions in the crystal structure of T1-Se-I.

In the crystal structure of **T1-Se-I**, a hydrogen-bond dimer, N13-H13A···N11 (Figure 8), is present along with a single N13-H13B···N12 hydrogen bond, which is similar to **T1-S-Br** and Form II of **T1-S-I**. Consistent with the interaction observed for Se in **T1-Se-Br**, the selenium atom of **T1-Se-I** also showed bifurcated chalcogen bonding, Se9···C3(π), at 3.487(4)Å and 165.75(14)°, and Se9···N12, at 3.613(3) Å and 154.88(13)°. In addition, the structure contains one Type II halogen bond, I7···I7, at 4.0468(4) Å and 162.8(1)°.

All relevant hydrogen, halogen and chalcogen bond geometries of T1-S-Br, T1-Se-Br, T1-S-I and T1-Se-I are given in Table 2.

## 3. Discussion

The calculated MEPs showed that when the smaller atoms (Br, S) were replaced by a more polarizable alternative (I, Se), the electrostatic potentials undergo significant transformations (Table 1, Figure 3). Changing bromine to iodine resulted in a 40% increase in the positive σ-hole potential, while a selenium replacement of sulfur produced a 23% enhancement. Even though intermolecular halogen bonds and chalcogen bonds are the result of contributions from electrostatic, polarization, repulsion, and dispersion, it is helpful to note that a relatively simplistic focus on the electrostatic component alone still offers valuable guidance for predicting which synthons are going to be more likely to appear in an extended structure. The increases in the electrostatic potential produce atoms that are more likely to form more prominent halogen and chalcogen bonds, respectively, which directly impacts their ability to more frequently deliver structure directing interactions in the solid state. 

**Figure 9:**
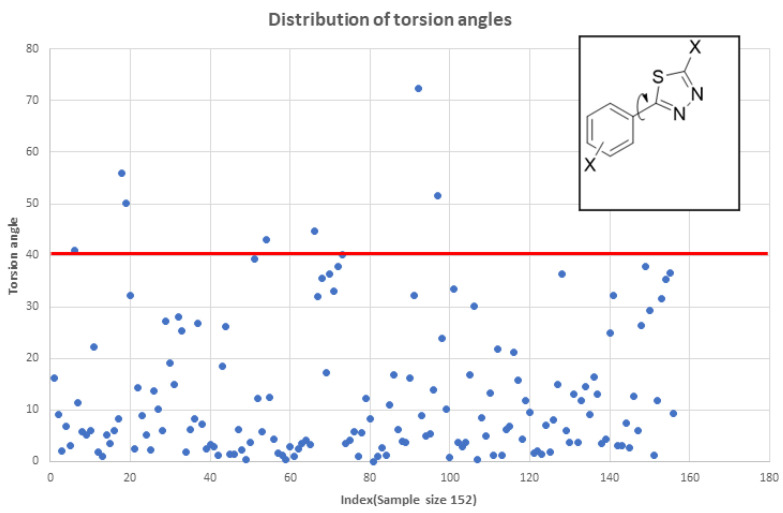
Distribution of torsion angles based on 152 CSD-reported 1,3,4-thiadiazol structures.

Geometry optimizations (in vacuo) of all target molecules showed the two rings, phenyl and 1,3,4-chalcogenadiazole, in each molecule to be close to coplanar geometry with a range of torsion angles of −0.55 to +0.55° (Figure S4). The planarity of these optimized structures only allows one of the two σ-holes to be accessed by the acceptors. In the solid-state, however, these were not planar and had torsion angles ranging from 30–40° (Figure S4). In the sulfur analogs of 1,3,4-chalcogendiazole published in the CSD (Figure 9, based on 152 relevant crystal structures), the majority had torsion angles less than 40°and most of them formed single chalcogen bond formation. The structures of the selenium analogues in this study contained bifurcated chalcogen bond comprising both σ-holes. As a result of the substitution of sulfur with selenium, an additional chalcogen bond becomes structurally active and the supramolecular assembly proceeds along a different path.

**Scheme 2:**
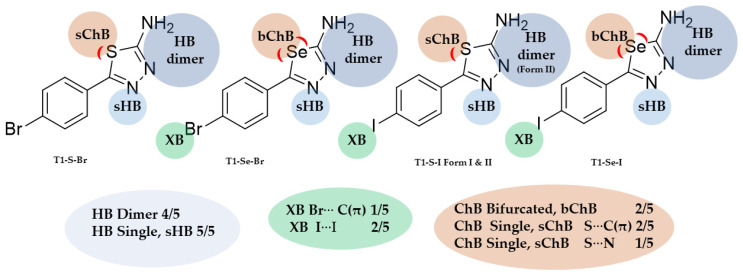
Summary of notable intermolecular interactions in the crystal structures examined herein. sHB: Single Hydrogen Bond. sChB: Single Chalcogen Bond. bChB: Bifurcated Chalcogen Bond.

Atom-to-atom replacement of halogen atoms resulted in a more significant enhancement of the electrostatic potentials compared to those noted in the case of the chalcogen replacement. Despite this difference, only 3/5 structures contained noteworthy halogen bonds, whereas chalcogen bonds were present in all five crystal structures examined herein. Only one of the two bromine-based compounds formed halogen bonds, whereas the iodine compounds showed Type II halogen bond formation in 2/5 structures, Scheme 2. All iodine-containing structures not undergoing halogen bonding show that despite the predictability of the MEPs, it was not always perfect; however, in general, the increased structural influence can be attributed to larger σ-hole potentials of the more polarizable iodine atom. The lack of halogen bonds in previously reported fluorine [38] and chlorine [39] analogs further validates the expectation that a more polarizable halogen atom is more likely to influence the solid-state assembly in ways to go beyond simple (and less directional) packing forces.

A comparison of the frequency of occurrence of hydrogen, halogen, and chalcogen bonds in the crystal structures of these 1,3,4-chalcogendiazole shows that the former was the most prominent; hydrogen bonding exists in all of them and 4/5 exist as hydrogen bond dimers and 5/5 as single hydrogen bond formation. The significance of the hydrogen bonding and the delicate balance between the hydrogen, halogen, and chalcogen bonding was clearly displayed in the two polymorphic structures. The melting points of Form I and Form II only differed by 1–2 °C, implying that their lattice energies (and relative stabilities) are closely matched. Since Form I contains halogen and hydrogen bonds (chalcogen bonding was overshadowed by the hydrogen bonding), and Form II contains chalcogen and hydrogen bonds, we can surmise that the impacts on the stability and physical properties of the two σ-hole interactions are very similar.

## 4. Materials and Methods

### 4.1. Reagent and General Methods

All reagents were used as received without any further purification. MEPs were calculated using density functional theory on molecules optimized in the gas phase using Spartan’ 08 program with B3LYP/6-311++G** level of theory. All the NMRs were recorded on a Bruker 400 MHz spectrophotometer. Single crystal X-ray diffraction data were obtained using a Bruker MicroStar and a Rigaku XtaLAB Synergy-S with CuK_α_ and MoK_α_ sources. The melting points were collected using a TA instrument DSC Q20 differential scanning calorimeter.

### 4.2. Crystallization Experiments

Single crystals of the pure products of **T1-Se-Br** and **T1-Se-I** were obtained using the slow evaporation method with methanol as the solvent. The pure product of **T1-S-I** was recrystallized using the slow evaporation method from acetonitrile and methanol to obtain Form I and Form II, respectively.

### 4.3. Synthesis of Selenosemicarbazide

Thiosemicarbazide (3.64 g, 0.04 mol) was dissolved in dry ethanol and to this, iodomethane (5.67 g, 0.04 mol) was added and the mixture was refluxed at 80 °C for 3 h. Upon cooling the mixture in an ice bath, the product, hydrazinyl(methylthio)methanamine, precipitated and this was air dried overnight and used for the following step.

To a mixture of Se and NaBH_4_ cooled to 0 °C with ice, dry ethanol was added under N_2_ atmosphere. Once all the Se dissolved, hydrazinyl(methylthio)methanamine dissolved in dry ethanol was added slowly. This was stirred overnight, and the resulting precipitate was filtered in the fume hood, and the product was purified through column chromatography as a grey/violet solid [31]. Yield 60%. Decomp: 174–176. ^1^H NMR (400 MHz, DMSO-*d*_6_) δ 9.07 (s, 1H), 7.97 (s, 1H), 7.64 (s, 1H), 4.52 (s, 2H), ^13^C NMR (101 MHz, DMSO) δ 175.80. ^77^Se NMR (400 MHz, DMSO-*d_6_*) δ 146.16.

### 4.4. Synthesis of 5-(4-Bromophenyl)-1,3,4-thiadiazol-2-amine, T1-S-Br

First, 4-bromobenzoic acid (2.01 g; 0.01 mol), thiosemicarbazide (0.91g, 0.01 mol), and POCl_3_ (5.0 mL) were mixed in a round-bottomed flask. This was refluxed at 75 °C for 3 h. After cooling to room temperature, water (50.0 mL) was added slowly to the mixture and the reaction mixture was further refluxed for 4 h. The progress of the reaction was monitored using TLC and after the completion, the mixture was cooled to room temperature and was basified to pH 8 using 30% NH_4_OH. The resulting precipitate was filtered, and the product was purified using column chromatography. The product was a light-yellow solid with a yield of 85%. MP: 212–214 °C. ^1^H NMR (400 MHz, DMSO-*d*_6_) δ 7.68 (q, *J* = 8.7 Hz, 4H), 7.50 (s, 2H). ^13^C NMR (101 MHz, DMSO) δ 169.32, 155.66, 132.55, 130.66, 128.60, 123.08.

### 4.5. Synthesis of 5-(4-Bromophenyl)-1,3,4-selenadiazol-2-amine, T1-Se-Br

First, 4-bromobenzoic acid (2.01g, 0.01 mol), selenosemicarbazide (1.38g, 0.01 mol), and POCl_3_ (5.0 mL) were mixed in a round-bottomed flask. This was refluxed at 75 °C for 3 h. After cooling to room temperature, water (50.0 mL) was added slowly to the mixture and the reaction mixture was further refluxed for 4 h. The progress of the reaction was monitored using TLC and after the completion, the mixture was cooled to room temperature and was basified to pH 8 using 30% NH_4_OH. The resulting precipitate was filtered, and the product was purified using column chromatography. The product was a light pink color solid with a yield of 65%. MP: 222–224 °C. ^1^H NMR(400 MHz, DMSO-*d_6_*) δ 7.64 (m, *J* = 8.5 Hz, 4H). ^13^C NMR (101 MHz, DMSO) δ 173.10, 160.83, 133.54, 132.42, 129.30, 122.91. ^77^Se NMR (400 MHz, DMSO-*d_6_*) δ 562.04.

### 4.6. Synthesis of 5-(4-Iodophenyl)-1,3,4-thiadiazol-2-amine, T1-S-I

First, 4-iodobenzoic acid (2.48g, 0.01 mol), thiosemicarbazide (0.91g, 0.01 mol), and POCl_3_ (5.0 mL) were mixed in a round-bottomed flask. This was refluxed at 75 °C for 3 h. After cooling to room temperature, water (50.0 mL) was added slowly to the mixture and the reaction mixture was further refluxed for 4 h. The progress of the reaction was monitored using TLC and after the completion, the mixture was cooled to room temperature and was basified to pH 8 using 30% NH_4_OH. The resulting precipitate was filtered, and the product was purified using column chromatography. The product was a white solid with a yield of 70%. MP: Form I 239–241 °C, From II 240–242 °C. ^1^H NMR (400 MHz, DMSO-*d_6_*) δ 7.83 (d, *J* = 8.5 Hz, 2H), 7.55 (d, *J* = 8.5 Hz 2H), 7.49 (s, 2H). ^13^C NMR (101 MHz, DMSO) δ 169.45, 156.11, 138.57, 131.16, 128.75, 96.62. 

### 4.7. Synthesis of 5-(4-Iodophenyl)-1,3,4-selenadiazol-2-amine, T1-Se-I

First, 4-iodobenzoic acid (2.48g, 0.01 mol), selenosemicarbazide (1.38g, 0.01 mol), and POCl_3_ (5.0 mL) were mixed in a round-bottomed flask. This was refluxed at 75 °C for 3 h. After cooling to room temperature, water (50.0 mL) was added slowly to the mixture and the reaction mixture was further refluxed for 4 h. The progress of the reaction was monitored using TLC and after the completion, the mixture was cooled to room temperature and was basified to pH 8 using 30% NH_4_OH. The resulting precipitate was filtered, and the product was purified using column chromatography. The product was a greyish solid with a yield of 55%. MP: 219–221 °C. ^1^H NMR (400 MHz, DMSO-*d*_6_) δ 7.80 (d, *J* = 8.4 Hz, 2H), 7.61 (s, 2H), 7.51 (d, *J* = 8.4 Hz, 2H). ^13^C NMR (101 MHz, DMSO) δ 173.00, 161.07, 138.27, 133.86, 129.30, 96.24. ^77^Se NMR (400 MHz, DMSO-*d_6_*) δ 560.97.

## 5. Conclusions

In summary, it is clear that atom-to-atom substitution can lead to significant changes in crystal structures especially when the less polarizable sulfur is replaced by a more polarizable selenium atom (a better chalcogen donor), leading to a more structurally influential σ-hole. Despite the fact that chalcogen bonds comprise a variety of components, MEPs offer a simple way of mapping out electrostatic potentials in the 1,3,4-chalcogenadiazole systems. This information can provide reliable guidelines for predicting which intermolecular interactions are going to be prominent in the solid state. By altering these electrostatic potentials through covalent means, the influence of σ-hole interactions (such as halogen and chalcogen bonds) can be controlled. Ultimately, this is of considerable practical importance when designing crystal engineering strategies that involve a combination of reversible non-covalent interactions.

## Figures and Tables

**Table 1:** MEP (kJ/mol) data for chalcogen and halogen atoms of the different target analogs.

Donor Atom	T1-S-Br	T1-Se-Br	T1-S-I	T1-Se-I
Chalcogen	96.6	118.8	95.7	117.5
Halogen	74.6	73.1	103.0	103.0

**Table 2:** Hydrogen, halogen, and chalcogen bond parameters of the five crystal structures.

	DH/X/Ch---A	D/X/Ch---A Å	DH/X/Ch---A(deg)
1-S-Br	N3-H1···N1	2.957	163.42
	N3-H2···N1	2.996	167.10
	S1···C5	3.497	161.20
T1-Se-Br	N33-H33b···N11	2.943(8)	165.3(2)
	N13-H13b···N31	2.942(8)	166.1(2)
	N33-H33a···N32	2.981(7)	157.05(19)
	N13-H13a···N12	2.976(7)	157.82(19)
	Se29···N32	3.646(5)	154.39(19)
	Se29···C25	3.416(6)	162.6(2)
	Se9···N12	3.603(6)	155.39(19)
	Se9-C3	3.406(6)	163.3(2)
	Br27···C4	3.517(6)	170.0(2)
T1-S-I Form I	N1B-H1BA···N3D	3.013(13)	176.8(7)
	N1A-H1AA···N3C	3.016(13)	179.2(7)
	N1C-H1CA···N3A	3.028(13)	179.2(7)
	N1D-H1DA···N3B	3.008(13)	178.9(8)
	N1C-H1CB···N2B	3.031(12)	157.4(6)
	N1A-H1AB···N2D	2.992(12)	155.5(6)
	N1D-H1DB···N2A	3.033(13)	158.7(13)
	N1B-H1BB···N2C	3.027(12)	159.5(6)
	S1C···N2B	3.507(9)	163.6(4)
	S1D···N1A	3.517(10)	164.6(4)
	S1A···N2D	3.525(10)	162.4(4)
	S1B···N2C	3.559(10)	163.1(4)
	I1C···I1A	3.892(1)	174.7(3)
	I1A···I1B	3.813(1)	176.3(3)
T1-S-I Form II	N6-H6B···N4	2.979(8)	178(8)
	N6-H6A···N5	3.019(7)	172(6)
	S2···C9	3.568(7)	162.1(2)
T1-Se-I	N13-H13A···N11	2.940(5)	165.6(3)
	N13-H13B···N12	2.957(4)	157.7(3)
	Se9···N12	3.613(3)	154.88(13)
	Se9···C3	3.487(4)	165.75(14)
	I7···I7	4.0468(4)	162.8(1)
	I7···I7	4.0468(4)	93.14(11)

## Data Availability

Crystallographic data (CCDC 2083577, 2083645, 2083656, 2083657) can be obtained free of charge via www.ccdc.cam.ac.uk/data_request/cif (accessed on 14 May 2021), or by emailing data_request@ccdc.cam.ac.uk, or by contacting The Cambridge Crystallographic Data Centre, 12 Union Road, Cambridge CB2 1EZ, UK; fax: +44 1223 336033.

## Supplementary Materials

The following are available online. Table S1: Hydrogen bond propensity (HBP) calculations using Mercury, Scheme S1: Schematic of 1,3,4-Chalcogenadiazole and the numbering used in HBP calculations, Figure S1: T1-Se-Br Secondary interactions of the second unique molecule of the asymmetric unit, Figure S2: T1-S-I Form I Halogen bonding interactions formed by each unique molecule in the asymmetric unit showing different bond geometries, Figure S3: T1-S-I Form I Hydrogen bonder 2-D chain, Figure S4: T1-S-Br a) torsion angle of the optimized structure in vacuum b) torsion angle of the molecule in the crystal structure, refcode XUVTAK, Table S2: Crystallographic data. ^1^H, ^13^C and ^77^Se NMR spectrums.

## Sample Availability

Samples of the compounds are not available from the authors.
