# Supplementary material for: The Balance between Hydrogen Bonds, Halogen Bonds, and Chalcogen Bonds in the Crystal Structures of a Series of 1,3,4-Chalcogenadiazoles
Source: Molecules. 2021 Jul 7;26(14):4125. doi: 10.3390/molecules26144125 (PMC8306955; doi:10.3390/molecules26144125)
Supplement: Supplementary file 1 [file molecules-26-04125-s001.zip › molecules-1250703-supplementary.pdf]

molecules

MDPI

Supporting Information

The balance between hydrogen bonds, halogen bonds, and chalcogen bonds in the crystal structures of a series of 1,3,4-chalcogenadizoles

Viraj De Silva[1], Boris B. Averkiev[1], Abhijeet S. Sinha[1] and Christer B. Aakeröy [1,*]

[1] Kansas State University, Department of Chemistry; aakeroy@ksu.edu
* Correspondence: aakeroy@ksu.edu; Tel.: +1-785 532 6096

Table S1 Hydrogen Bond Propensity(HBP) calculations using Mercury.

Target	DH···A	HBP
T1-S-Br	N1H···N2	0.93
	N1H···N3	0.91
	N1H···Br	0.10
	N1H···S1	0.03
T1-Se-Br	N1H···N2	0.85
	N1H···N3	0.82
	N1H···Br	0.08
T1-S-I	N1H···N2	0.95
	N1H···N3	0.93
	N1H···I	0.20
	N1H···S	0.03
T1-Se-I	N1H···N2	0.93
	N1H···N3	0.91
	N1H···I	0.20

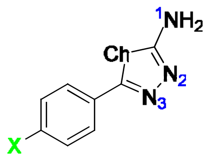

Scheme S1. Schematic of 1,3,4-Chalcogendiazole and the numbering used in HBP calculations.

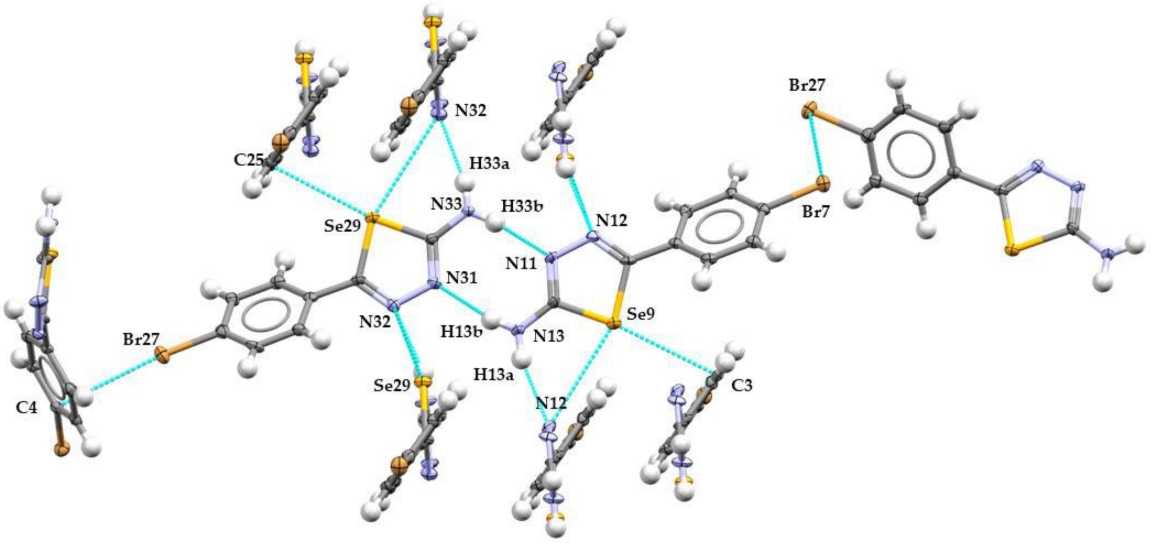

Figure S1 T1-Se-Br Secondary interactions of the second unique molecule of the asymmetric unit.

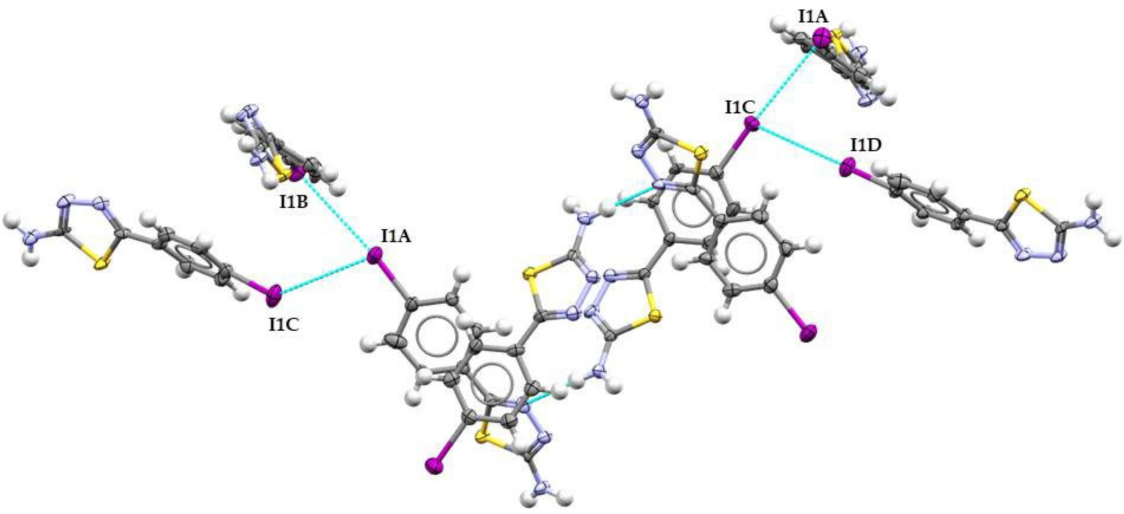

Figure S2. T1-S-I Form I Halogen bonding interactions formed by two unique molecules in the asymmetric unit showing different bond geometries.

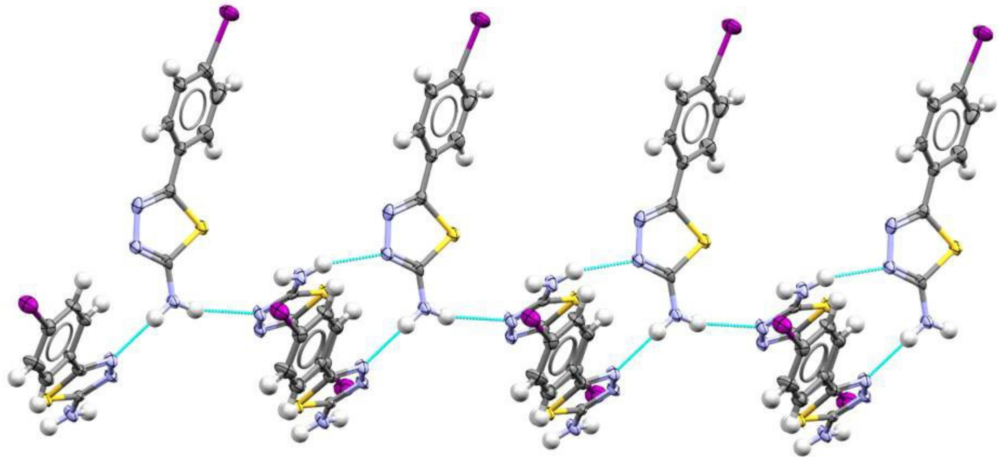

Figure S3. T1-S-I Form I Hydrogen bonder 2D chain.

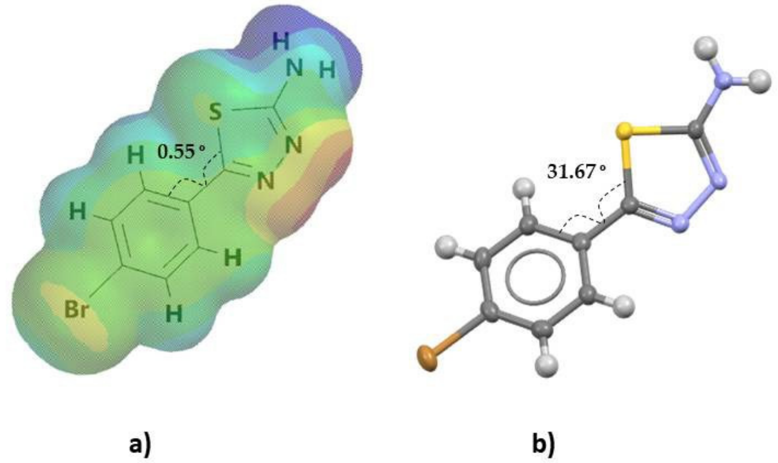

Figure S4. T1-S-Br a) torsion angle of the optimized structure under vacuume b) torsion angle of the crystal structure, refcode XUVTAK.

Table S2. Crystallographic data

Code	**T1-Se-Br**	**T1-S-I Form I**	**T1-S-I Form II**	**T1-Se-I**
Formula moiety	2(C_8 H_6 Br N_3 Se)	$C_8H_6IN_3S$	C_8 H_6 I N_3 S	$C_8H_6IN_3Se$
Empirical formula	$C_{16}H_{12}Br_2N_6Se_2$	$C_8H_6IN_3S$	C_8 H_6 I N_3 S	$C_8H_6IN_3Se$
Molecular weight	606.040	303.12	303.12	350.02
Color, Habit	Yellow, Needle	Colorless, Plate	Colorless, Rectangular	Colorless, Rectangular
Crystal system	orthorhombic	monoclinic	monoclinic	monoclinic

Space group, Z	$Pca2_1$	C1c1	P 1 21/c 1	$P2_1/c$
a, Å	10.9000(2)	12.00353(18)	12.7804(6)	15.5622(3)
b, Å	5.8066(1)	11.89672(19)	7.3454(4)	5.96390(10)
c, Å	29.4257(4)	27.1321(4)	11.2053(5)	10.9214(2)
α, º	90	90	90	90
β, º	90	95.8033(14)	109.268(2)	98.3760(10)
γ, º	90	90	90	90
Volume, $Å^3$	1862.41(5)	3854.68(11)	993.00(8)	1002.82(3)
Density, g/cm^3	2.161	2.089	2.028	2.318
T, ºK	100.00(10)	200.00(10)	199.99	199.99
Crystal size, min x mid x max	0.10 × 0.04 × 0.02	0.16 × 0.1 × 0.03	0.17 × 0.06 × 0.04	0.05 × 0.03 × 0.02
X-ray wavelength, Å	1.54184	0.71073	1.54178	1.54178
μ, mm^{-1}	10.112	3.494	26.954	28.895
Trans min / max	0.06603, 1.00000	0.49396, 1.00000	0.3420, 0.7531	0.326, 0.596
θ_{min}, º	3.00	2.417	3.664	2.870
θ_{max}, º	77.39	27.999	68.442	70.232
Reflections				
collected	15890	25434	5638	7588
independent	3626	8170	1756	1846
observed	3517	7479	1571	1771
R_{int}	0.0559	0.0215	0.0528	0.0339
Threshold expression	$> 2\sigma(I)$	$> 2\sigma(I)$	$> 2\sigma(I)$	$> 2\sigma(I)$
No. parameters	236	470	126	119
No. restraints	1	8	0	0

R_1 (observed)	0.0373	0.0436	0.0435	0.0284
wR_2 (all)	0.1016	0.1108	0.128	0.0722
Goodness of fit (all)	1.0357	1.148	1.093	1.077
ρ_{max}, ρ_{min}, e Å^{-3}	0.9954, -0.8715	2.589, -0.857	0.808, -1.157	1.405, -0.700
Completeness to 2θ limit	0.9703	0.985	0.959	0.967

Referencess

1. Wood, P.A.; Feeder, N.; Furlow, M.; Galek, P.T.A.; Groom, C.R.; Pidcock, E. Knowledge-based approaches to co-crystal design. *CrystEngComm* **2014**, *16*, 5839–5848, doi:10.1039/c4ce00316k.
